# Inhibition of glycogen synthase kinase-3β is involved in cardioprotection by α7nAChR agonist and limb remote ischemic postconditionings

**DOI:** 10.1042/BSR20181315

**Published:** 2018-10-15

**Authors:** Hui-Xian Li, Xin-Long Cui, Fu-Shan Xue, Gui-Zhen Yang, Ya-Yang Liu, Qing Liu, Xu Liao

**Affiliations:** Department of Anesthesiology, Plastic Surgery Hospital, Chinese Academy of Medical Sciences and Peking Union Medical College, Beijing, China

**Keywords:** Cholinergic anti-inflammatory pathway, Glycogen synthase kinase-3β, Limb remote ischemic postconditioning, Myocardial ischemia/reperfusion injury

## Abstract

The present study was designed to determine whether glycogen synthase kinase-3β (GSK-3β) was involved in the cardioprotection by α7 nicotinic acetylcholine receptor (α7nAChR) agonist and limb remote ischemic postconditionings. Forty male Sprague-Dawley rats were randomly divided equally into control (C), α7nAChR agonist postconditioning (P), limb remote ischemic postconditioning (L), combined α7nAChR agonist and limb remote ischemic postconditioning (P+L) groups. At the end of experiment, serum cTnI, creatine kinase-MB (CK-MB), tumor necrosis factor-α (TNF-α), interleukin-6 (IL-6), high mobility group protein (HMGB1) and interleukin-10 (IL-10) levels were measured; infarct size (IS), myocardial expressions of GSK-3β, p-GSK-3β (Ser9), nuclear factor-κB (NF-κB) and p-NF-κB (Ser536) in the ischemic area were assessed. The results showed that compared with group C, IS, serum cTnI and CK-MB levels obviously decreased in groups P, L and P+L. Compared with groups P and L, IS, serum cTnI and CK-MB levels significantly decreased in group P+L. Compared with group C, serum TNF-α, IL-6 and HMGB1 levels, and myocardial expression of p-NF-κBp65 (Ser536) evidently decreased, and myocardial expression of p-GSK-3β (Ser9) obviously increased in groups P, L and P+L. Compared with group P, serum TNF-α, IL-6 and HMGB1 levels and myocardial expression of p-NF-κBp65 (Ser536) significantly increased, and myocardial expression of p-GSK-3β (Ser9) evidently decreased in group L. Compared with group L, serum TNF-α, IL-6, HMGB1 levels, and myocardial expression of p-NF-κBp65 (Ser536) significantly decreased, and myocardial expression of p-GSK-3β (Ser9) obviously increased in group P+L. In conclusion, our findings indicate that inhibition of GSK-3β to decrease NF-κB transcription is one of cardioprotective mechanisms of α7nAChR agonist and limb remote ischemic postconditionings by anti-inflammation, but improved cardioprotection by combined two interventions is not completely attributable to an enhanced anti-inflammatory mechanism.

## Introduction

The available evidence shows that inflammation plays a pivotal role in the mechanisms of ischemia reperfusion injury (IRI) and regulating inflammatory response can provide a protection against myocardial IRI [1]. The cholinergic anti-inflammatory pathway (CAP), which is consisted of vagus nerve, acetylcholine and α7 nicotinic acetylcholine receptor (α7nAChR), is an immunomodulatory mechanism [1–3]. It has been demonstrated that postconditioning with α7nAChR agonist can significantly attenuate myocardial IRI by inhibiting inflammatory responses via the CAP [4]. As a safe and effective cardioprotective intervention, moreover, limb remote ischemic postconditioning has been shown to provide a significant protection against myocardial IRI by attenuating regional and systemic inflammatory responses [5]. However, mechanisms of above two postconditionings to provide cardioprotection by modulating inflammatory response have not been fully clarified.

Glycogen synthase kinase-3β (GSK-3β) is a widely existing serine threonine kinase, and can modulate many factors mediating inflammatory response and affect activity of nuclear factor-κB (NF-κB), which is a key regulator of the inflammatory response [6,7]. Mostly important, it has been shown that GSK-3β is involved in the physiopathological mechanisms of myocardial IRI and is attributable to cardioprotection of many interventions [8–10]. Thus, we speculate that GSK-3β may be involved in cardioprotective mechanisms of postconditioning with α7nAChR agonist and limb remote ischemia. The purpose of this experiment was to determine whether GSK-3β was involved in cardioprotection by α7nAChR agonist and limb remote ischemic postconditionings via anti-inflammatory mechanism.

## Materials and methods

### Surgical preparation of animals

After the experimental protocol was approved by the Animal Care and Use Committee of Plastic Surgery Hospital, Chinese Academy of Medical Sciences, the healthy male Sprague-Dawley rats aged 8 weeks and weighting 290–320 g were used in the present study. All animals were provided by the Beijing Vital River Laboratory Animal Technology Company. All the rats were acclimatized to the environment with food and water available *ad libitum* for 2 weeks before experiment. They were housed in a quiet, temperature (24 ± 1°C) and humidity (65 ± 10%) controlled room with a 12-h:12-h light–dark cycle (light beginning at 8 a.m.), and all experiments were performed during the light phase of the cycle. Before experiment, animal was fasted for 12 h, but drunk freely.

The rat model of acute myocardial IRI was established, as previously described [11]. After adequate anesthesia with intraperitoneal injection of sodium pentobarbital and tracheal intubation, the rat was ventilated with room air using an animal respirator. The ventilation rate was adjusted to 60–80 breaths/min, with the tidal volume of 2–3 ml/100 g body weight and the inspiratory/expiratory ratio of 1:1. The internal jugular vein was cannulated for blood sampling to assay serum concentrations of troponin I (TnI), creatine kinase-MB (CK-MB), and inflammatory cytokines including tumor necrosis factor-α (TNF-α), interleukin-6 (IL-6), high mobility group protein (HMGB1) and interleukin-10 (IL-10). The carotid artery was cannulated for monitoring heart rate, systolic blood pressure, diastolic blood pressure and mean artery pressure with a MP150 data acquisition and analysis system (Biopac Systems Inc., CA, U.S.A.). The lead II electrocardiogram was continuously recorded by the means of needle electrodes placed subcutaneously on the limbs.

A left thoracotomy was performed via the fourth intercostal space, and the left anterior wall and auricle of the heart were exposed. After pericardiotomy, a 5-0 silk ligature was placed under the left anterior descending coronary artery (LAD). After an equilibration of 10 min, the ligature was tied for 30 min to block blood flow of LAD and then relaxed for 120 min to resume blood flow of LAD, producing a local myocardial IRI.

### Experimental protocols

By a random number table, 40 rats in whom acute myocardial IRI model had been successfully established were randomly divided equally into four groups subjected to different protocols (10 per group), i.e. control (C), α7nAChR agonist postconditioning (P), limb remote ischemic postconditioning (L) and combined two interventions (P+L) groups. All the rats received the thoracotomy and a 30-min ligature of LAD for ischemia followed by a 120-min reopening of LAD for reperfusion *in vivo*. In the group C, no additional intervention was performed. In the group P, PNU282987, a specific α7nAChR agonist, was intravenously injected immediately before reperfusion. The dosage of PNU282987 was 2 mg/kg and it was diluted with 1 ml saline before use. In the group L, after 20 min of LAD ligature, blood flow in the bilateral hind limbs were stopped for 10 min and then opened with reperfusion of LAD simultaneously. In the group P+L, the rats received the same protocols as those of the groups P and L.

### Observed variables

#### Measurement of serum cTnI and CK-MB levels

At 120 min of reperfusion, 1-ml blood sample was collected in the tubes containing EDTA from the right internal jugular veins. After setting for 30 min, the blood sample was centrifuged at 2500 rpm for 10 min with 12-cm radius. The supernatant was collected and stored in an EP tube at −80 °C condition until subsequent analysis. The serum cTnI and CK-MB levels were assessed respectively by enzyme-linked immunosorbent assay (ELISA) kits specifically for rats. The specific steps were carried out in accordance with manufacture’s instruction (Rapidbio, West Hills, CA, U.S.A.).

#### Infarct size evaluation

After a 120-min reperfusion, the LAD was re-occluded and 2–3 ml of 2% Evans blue dye was injected into the carotid artery to stain the normally perfused region of the heart and delineate the area at risk (AAR). Then the rat was killed using an intravenous injection of 10% potassium chloride. The entire heart was excised and the whole atria and right ventricle were removed. The remaining left ventricle was cut into 4–5 sections from apex to base perpendicularly to the ventricle. The area stained with blue in the section was the normally perfused region of the heart and the unstained area was delineated as AAR. Then all tissues were incubated in the 1% solution of 2,3,5-triphenyltetrazolium chloride PBS for 10–15 min at 37°C. The viable tissue stained red, whereas the infarcted tissue stained characteristic white. The analysis of the sections was conducted by Image-Pro Plus 5.0 to assess the percentage of infarct size and AAR to total area, which was used to calculate the infarct size (IS).

#### Measurement of serum inflammatory cytokines

At 120 min of reperfusion, 1-ml blood sample was collected into the tubes containing microscopic silica particles. Blood samples were centrifuged at 3000 rpm for 15 min. Supernatants were obtained and stored at −80°C condition for future analysis. A blinded investigator assayed serum TNF-α, IL-6, HMGB-1 and IL-10 levels using enzyme-linked immunosorbent assay (ELISA) kits according to the manufacturer’s instructions (Rapidbio, West Hills, CA, U.S.A.).

#### Myocardial expressions of GSK-3β and NF-κB by Western blotting

To detect the expressions of GSK-3β and NF-κB in the ischemic myocardium, left ventricle was quickly removed after 120 min of reperfusion and stored at −20°C condition. The myocardial tissues were cut into small pieces and the total protein of each sample was extracted. Then, protein concentration was measured by BCA assay. Equal amounts of protein (50 μg) in each group were electrophoresed (SDS-PAGE, 10% of separation gel, 5% of concentration gel) and transferred to nitrocellulose membrane. After blocking and eluting, the membranes were incubated overnight shaking at 4°C with monoclonal antibodies GSK-3β (1:1000), p-GSK-3β Ser9 (1:1000), NF-κBp65 (1:1000), p-NF-κBp65 Ser536 (1:1000) and β-actin (1:1000), respectively. Then the membranes were cleaned and incubated with horseradish peroxidase-conjugated second antibody (1:5000) for 1 h at room temperature. The antigen–antibody complexes in the membranes were visualized using enhanced chemiluminescence (ECL) and films were exposed in the darkroom. The time of exposure, development and fixing were dependent on the darkness of bands. Then, the films were scanned and saved as TIF image files. The band intensity was quantified using Quantity One Analysis Software. Finally, expressions of proteins were acquired by standardizing the grey levels of GSK-3β, p-GSK-3β Ser9, NF-κBp65 and p-NF-κBp65 Ser536 with β-actin.

### Statistical analysis

Statistical analysis of data was completed with SPSS (Version 18.0, SPSS, Inc., Chicago, IL, U.S.A.). All measurement data were tested for normality using Shapiro–Wilk test and tested for homogeneity of variance using Levene test. If the data satisfied the normal distribution and homoscedasticity, they were presented as mean ± SD. One-way analysis of variance (ANOVA) was applied for comparison between groups. *P*<0.05 was considered statistically significant.

## Results

### Infarct size and serum c-TnI and CK-MB levels

**Figure 1:**
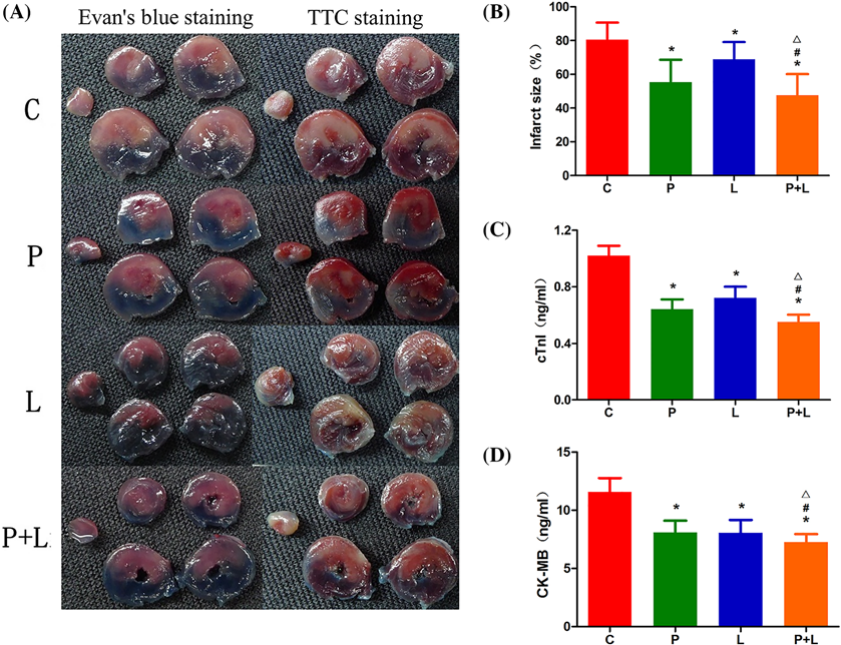
The infarct size, and serum c-TnI and CK-MB levels in the four groups. The typical stained myocardial slices (**A**), infarct size as a percentage of the area at risk (**B**), serum cTnI (**C**) and CK-MB (**D**) levels in the control (C), α7nAChR agonist postconditioning (P), limb remote ischemic postconditioning (L), combined α7nAChR agonist and limb remote ischemic postconditioning (P+L) groups. Data are presented as mean ± SD; *n* = 10 in each group. *P<0.05 compared with group C; ^#^*P*<0.05 compared with group P; ^△^*P*<0.05 compared with group L.

The infarct size was 80.5 ± 10.1%, 55.4 ± 13.2%, 68.9 ± 10.2% and 47.6 ± 12.5% in the groups C, P, L and P+L, respectively. Compared with the group C, infarct size significantly decreased in the groups P, L and P+L. The infarct sizes in the groups P and L were not evidently different, but were obviously larger than that in the group P+L (Figure 1A,B).

The serum c-TnI levels were 1.02 ± 0.07, 0.64 ± 0.07, 0.72 ± 0.08 and 0.55 ± 0.05 ng/ml in the groups C, P, L and P+L, respectively. The serum CK-MB levels were 11.6 ± 1.2, 8.1 ± 1.0, 8.1 ± 1.1 and 7.2 ± 0.7 ng/ml in the groups C, P, L and P+L, respectively. Compared with the group C, serum c-TnI and CK-MB levels significantly decreased in the groups P, L and P+L. The serum c-TnI and CK-MB levels in the groups P and L were not obviously different, but were evidently higher than that in the groups P+L (Figure 1C,D).

### Serum TNF-α, IL-6, HMGB1 and IL-10 levels

The serum TNF-α level was 762 ± 84, 76 ± 15, 211 ± 62 and 82 ± 12 pg/ml in the groups C, P, L and P+L, respectively. The serum IL-6 level was 756 ± 89, 491 ± 82, 644 ± 79 and 431 ± 67 pg/ml in the groups C, P, L and P+L, respectively. The serum HMGB1 level was 10.4 ± 1.2, 1.1 ± 0.1, 5.8 ± 0.4 and 1.2 ± 0.2 ng/ml in the groups C, P, L and P+L, respectively.The serum IL-10 level was 4.6 ± 1.0, 4.3 ± 1.2, 3.9 ± 0.6 and 4.0 ± 1.1pg/ml in the groups C, P, L and P+L, respectively.

**Figure 2:**
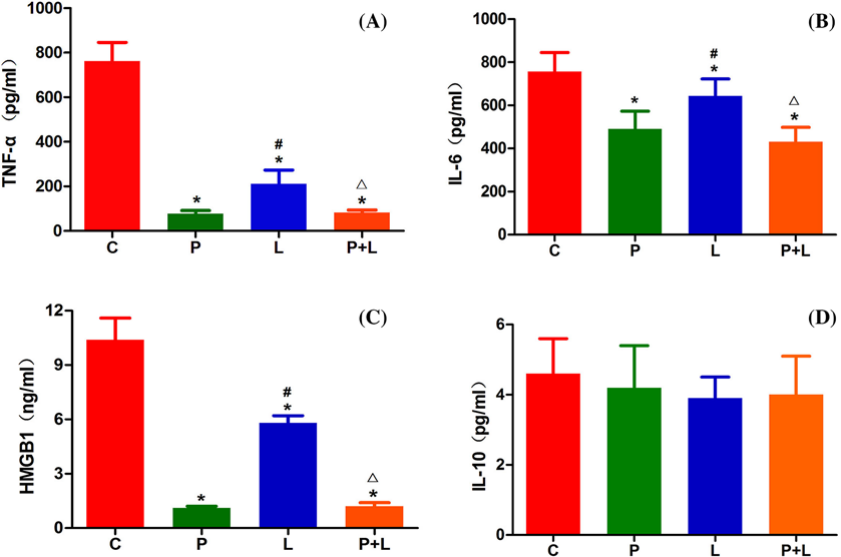
The serum levels of inflammatory cytokines in the four groups. The serum TNF-α (**A**), IL-6 (**B**), HMGB1 (**C**) and IL-10 (**D**) levels in the control (C), α7nAChR agonist postconditioning (P), limb remote ischemic postconditioning (L), combined α7nAChR agonist and limb remote ischemic postconditioning (P+L) groups. Data are presented as mean ± SD; *n* = 10 in each group. *P<0.05 compared with group C; ^#^*P*<0.05 compared with group P; ^△^*P*<0.05 compared with group L.

Compared with the group C, serum TNF-α, IL-6 and HMGB1 levels significantly decreased in the groups P, L and P+L. Compared with the group P, serum TNF-α, IL-6 and HMGB1 levels obviously increased in the group L, but did not evidently change in the group P+L. Compared with the group L, serum TNF-α, IL-6 and HMGB1 levels significantly decreased in the group P+L (Figure 2A–C). There was no obvious difference in serum IL-10 level among groups (Figure 2D).

### Myocardial expressions of GSK-3β and NF-κB

**Figure 3:**
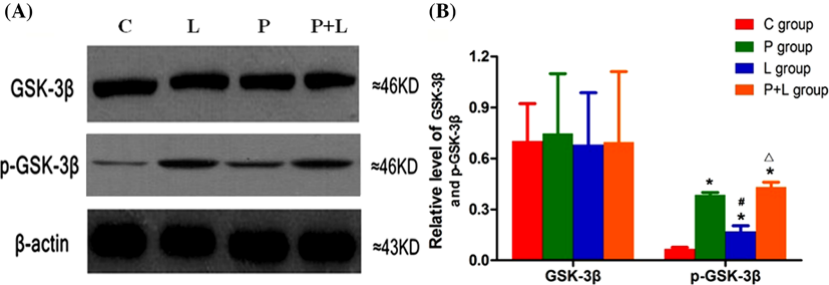
The myocardial expression of GSK-3β in the four groups. Myocardial expressions of GSK-3β and phosphorylated GSK-3β by Western blotting in the control (C), α7nAChR agonist postconditioning (P), limb remote ischemic postconditioning (L), combined α7nAChR agonist and limb remote ischemic postconditioning (P+L) groups. (**A**) Western blotting; (**B**) quantitative analysis for myocardial expressions of GSK-3β and phosphorylated GSK-3β. Data are presented as mean ± SD; *n* = 10 in each group. *P<0.05 compared with group C; ^#^*P*<0.05 compared with group P; ^△^*P*<0.05 compared with group L.

The variance analysis showed that there was no significant difference among groups in myocardial expression of GSK-3β in the ischemic area, but myocardial expression of p-GSK-3β (Ser9) was obviously different among groups. Compared with the group C, myocardial expression of p-GSK-3β (Ser9) significantly increased in the groups P, L and P+L. Compared with the group P, myocardial expression of p-GSK-3β (Ser9) obviously decreased in the group L, but did not evidently change in the group P+L. Compared with the group L, myocardial expression of p-GSK-3β (Ser9) obviously increased in the group P+L (Figure 3).

**Figure 4:**
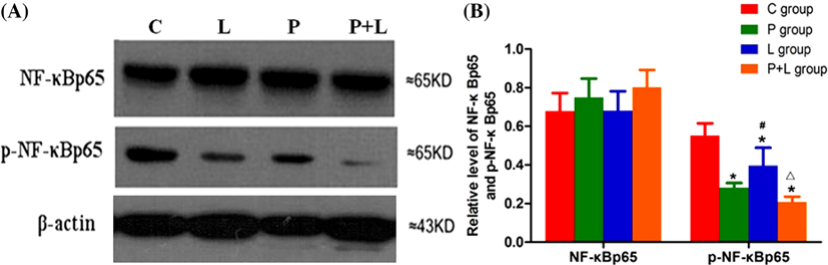
The myocardial expression of NF-κB in the four groups. Myocardial expressions of NF-κBp65 and phosphorylated NF-κBp65 (Ser536) in the control (C), α7nAChR agonist postconditioning (P), limb remote ischemic postconditioning (L), combined α7nAChR agonist and limb remote ischemic postconditioning (P+L) groups. (**A**) Western blotting; (**B**) Quantitative analysis for myocardial expressions of NF-κBp65 and phosphorylated NF-κBp65 (Ser536). Data are presented as mean ± SD. *n* = 10 in each group. *P<0.05 compared with group C; ^#^*P*<0.05 compared with group P; ^△^*P*<0.05 compared with group L.

The variance analysis showed that there was no significant difference among groups in myocardial expression of NF-κBp65 in the ischemic area, but myocardial expression of p-NF-κB p65 (Ser536) was evidently different among groups. Compared with the group C, myocardial expression of p-NF-κBp65 (Ser536) significantly decreased in the groups P, L and P+L. Compared with the group P, myocardial expression of p-NF-κBp65 (Ser536) obviously increased in the group L, but did not evidently change in the group P+L. Compared with the group L, myocardial expression of p-NF-κBp65 obviously decreased in the group P+L (Figure 4).

## Discussion

GSK-3β, a multifunctional protein kinase, is involved in the cell differentiation, proliferation, survival and apoptosis by identifying and phosphorylating the substrates with specific amino sequences [12]. In the molecular structure of GSK-3β, there are two major phosphorylation sites: Tyr216 and Ser9. Phosphorylation of Tyr216 site increases GSK-3β activity [13], while phosphorylation of Ser9 site forms a competitive site of substrate that results in decreased GSK-3β activity [14]. Our results showed that there was no significant difference in myocardial expression of GSK-3β in the ischemic area among groups. This is consistent with the finding of Wang et al’s study [11], in which an *in vivo* rat model of 30-min ischemia and 180-min reperfusion is also used and myocardial expression of GSK-3β is relatively stable before and after myocardial IRI process. This is probably because there is a stable expression of GSK-3β in myocardium and the interventions used in the present study have no significant effect on myocardial expression of GSK-3β.

However, this experiment showed that compared with the group C, myocardial expression of p-GSK-3β (Ser9) obviously increased in the groups P, L and P+L. It is in accord with previous findings of Tamareille et al’s study [15], in which an *in vivo* rat model of myocardial IRI is applied, infarct size significantly decreased and myocardial expression of p-GSK-3β (Ser9) evidently increased by limb remote ischemic postconditioning at 20 min of ischemia. These results suggest that inhibition of GSK-3β activity by enhancing phosphorylation of Ser9 site may be one of cardioprotective mechanisms of limb remote ischemic postconditioning. Moreover, our experiment first confirms that α7nAChR agonist postconditioning can also provide a significant protection against myocardial IRI by this pathway.

The available evidence shows that inflammatory hyper-responsiveness is a characteristic of IRI, which is basically induced by an endogenous mechanism called danger-associated molecular patterns (DAMPs) [16–18]. The DAMPs are mainly mediated by the Toll-like receptors [19]. When DAMPs are combined with the Toll-like receptors, they can activate NF-kB and P38, which lead to the expression and release of proinflammatory cytokines, such as TNF-α, IL-1, IL-6 and HMGB1. Then, proinflammatory cytokines active inflammatory cells, which can directly damage the cardiomyocytes [20]. Our experiment demonstrated that in the group C, ischemia–reperfusion was indeed associated with significantly inflammatory response and induced severe myocardial injury.

NF-κB is a key component of inflammatory response and immunoregulation [21,22]. NF-κB activation can induce production of proinflammatory cytokines by enhancing the transcription of related genes, such as TNF-α, IL-1, IL-6 and HMGB1. Importantly, newly produced proinflammatory cytokines can further activate NF-kB. This undesirable inflammatory cycle by positive feedback can enhance regional and systemic inflammatory responses. It is generally believed that proinflammatory cytokines including TNF-α and IL-6 have the adverse effects on the contractile function of cardiomyocytes and induce ventricular remodeling, which can worse the prognosis of damaged myocardium [23]. Moreover, GSK-3β can also affect activity of NF-κB. By using a mice model of GSK-3β gene deletion, Hoeflich et al. [24] demonstrate that GSK-3β has an important regulatory role in activation of NF-κB, evidenced by disappearance of the function that NF-κB induces cell differentiation. Schwabe et al. [25] find that GSK-3β can activate the transcriptional activity of NF-κB by phosphorylation of its p65 subunit. However, lithium chloride, an inhibitor of GSK-3β, can reduce production of inflammatory cytokines by decreasing NF-κB activity [26]. The present study showed that myocardial ischemia–reperfusion resulted in significant inflammatory responses in the group C, but α7nAChR agonist postconditioning, limb remote ischemic postconditioning and combined two interventions significantly reduced myocardial expression of NF-κB in the ischemic area, with decreased serum proinflammatory cytokine levels and infract sizes. According to the findings of previous and our studies, we consider that inflammatory inhibition, as an important cardioprotective mechanism of α7nAChR agonist and limb remote ischemic postconditionings [4,5], is achieved by decreasing NF-κB transcription through inhibition of GSK-3β activity. In the rats subjected to intestinal IRI, Cuzzoctea et al. [27] demonstrate that application of GSK-3β inhibitor (TDZD-8) before reperfusion can significantly inhibit the expression of p-NF-κBp65 and decrease serum levels of TNF-α and IL-6. These results support our speculation.

This experiment also showed that α7nAChR agonist postconditioning provided the stronger inhibitive effects on activities of GSK-3β and NF-κB than limb remote ischemic postconditioning, but infarct size was not significantly different between two interventions. This is probably because cardioprotection by α7nAChR agonist postconditioning mainly relies on anti-inflammatory effect [4,28], whereas cardioprotection by limb remote ischemic postconditioning is also dependent on other pathways [29]. Furthermore, combined two interventions provided an improved cardioprotection, but inflammatory responses were not obviously different between groups P and P+L. These results suggested that improved cardioprotection by combined two interventions was not completely attributable to an enhanced inflammatory inhibition by inhibiting GSK-3β activity. This provides further evidence that combined interventions may produce cardioprotection by different mechanisms. Other than inflammation inhibition, it has been shown that JAK/STAT and PI3K/Akt signaling pathways, JAK/STAT3-mediated Nrf2-antioxidant signaling pathway, endothelial nitric oxide synthase, antiapoptosis, mitochondrial permeability transition pore and ATP-sensitive potassium channel are involved in the cardioprotection by limb remote ischemic postconditioning [29–34]. Because there are the extensive cross interactions among these modulators and signaling pathways, enhanced cardioprotection can be achieved by combined different interventions.

Differing from proinflammatory cytokines including TNF-α, IL-6 and HMGB1, IL-10 is a pleiotropic anti-inflammatory cytokine that can inhibit macrophage activation and the subsequent production and release of proinflammatory cytokines [35]. An interesting finding of this study was that there was no significant difference in serum IL-10 level among groups. The possible reasons of this result include: (a) IL-10 is not involved in CAP [36], and (b) release of IL-10 into blood begins after 3 h of reperfusion and reaches the peak at 24–96 h of reperfusion [37]. In our experiment, serum samples were collected after 120 min of reperfusion. At this time point, thus, production and release of IL-10 cannot be measured. This finding suggests that a prolonged observation time is needed when serum IL-10 level is used to assess anti-inflammatory response during myocardial ischemia–reperfusion process.

In our experiment, there are some design limitations that deserve special attention. First, given the facts that the total blood volume of a rat is very small, and excessive blood loss from repeated blood samples may significantly affect hemodynamics and myocardial blood perfusion, serum levels of biomarkers were only measured at the end of reperfusion. The use of one time point to measure these biomarkers is inability to depict the panorama of myocardial injury and systemic inflammatory response. Second, the nuclear translocation of NF-kBp65 is important for the activation of NF-κB. This experiment assessed total expression of NF-κBp65 in the ischemic myocardium, but did not separate its cytosolic and nuclear fraction to show the translocation of NF-kBp65 in the nucleus. Thus, our findings may only reflect the total activation of NF-κB in the ischemic myocardium, but not the translocation of NF-κBp65 in the nucleus. Recently, other investigators had also used only p-NF-κBp65 to indicate the activation of NF-κB. Recently, other investigators had also used only pNF-κBp65 to indicate the activation of NF-κB [38,39]. Third, this experiment only assessed the protective mechanisms of α7nAChR agonist and limb remote ischemic postconditionings against myocardial IRI by modulating inflammatory response. Other than anti-inflammatory mechanism by inhibiting GSK-3β, available evidence shows that other factors mentioned above are also attributable to the cardioprotection induced by limb remote ischemic postconditioning [29–34]. Evidently, this experiment cannot provide any clue for the roles of these factors in the cardioprotection induced by limb remote ischemic postconditioning. These issues remain to be demonstrated by further researches.

## Conclusions

Our experiment demonstrates that inhibition of GSK-3β activity to decrease NF-κB transcription is one of cardioprotective mechanisms of α7nAChR agonist and limb remote ischemic postconditionings by anti-inflammation, but improved cardioprotection by combined two interventions is not completely attributable to an enhanced inflammatory inhibition.

## Abbreviations

α7nAChR: α7 nicotinic acetylcholine receptor
AAR: area at risk
CAP: cholinergic anti-inflammatory pathway
CK-MB: creatine kinase-MB
DAMP: danger-associated molecular pattern
GSK-3β: glycogen synthase kinase-3β
HMGB1: high mobility group protein
IL: interleukin
IRI: ischemia reperfusion injury
NF-κB: nuclear factor-κB
TNF-α: tumor necrosis factor-α
